# Influences of social uncertainty and serotonin on gambling decisions

**DOI:** 10.1038/s41598-022-13778-x

**Published:** 2022-06-17

**Authors:** Gabriele Bellucci, Thomas F. Münte, Soyoung Q. Park

**Affiliations:** 1 Department of Computational Neuroscience, Max Planck Institute for Biological Cybernetics, 72076 Tübingen, Germany; 2 Department of Psychology I, University of Lübeck, Lübeck, Germany; 3 Department of Neurology, Universitätsklinikum Schleswig-Holstein, Lübeck, Germany; 4 Department of Psychology II, University of Lübeck, Lübeck, Germany; 5 Decision Neuroscience and Nutrition, German Institute of Human Nutrition (DIfE), Potsdam-Rehbruecke, Nuthetal, Germany; 6 Neuroscience Research Center, Berlin Institute of Health, Corporate Member of Freie Universität Berlin, Charité-Universitätsmedizin Berlin, Humboldt-Universität zu Berlin, Berlin, Germany; 7 Deutsches Zentrum für Diabetes, Neuherberg, Germany

**Keywords:** Psychology, Human behaviour

## Abstract

In many instances in life, our decisions’ outcomes hinge on someone else’s choices (i.e., under social uncertainty). Behavioral and pharmacological work has previously focused on different types of uncertainty, such as risk and ambiguity, but not so much on risk behaviors under social uncertainty. Here, in two different studies using a double-blind, placebo-controlled, within-subject design, we administrated citalopram (a selective-serotonin-reuptake inhibitor) to male participants and investigated decisions in a gambling task under social and nonsocial uncertainty. In the social condition, gamble outcomes were determined by another participant. In the nonsocial condition, gamble outcomes were determined by a coin toss. We observed increased gamble acceptance under social uncertainty, especially for gambles with lower gains and higher losses, which might be indicative of a positivity bias in social expectations in conditions of high uncertainty about others’ behaviors. A similar effect was found for citalopram, which increased overall acceptance behavior for gambles irrespective of the source of uncertainty (social/nonsocial). These results provide insights into the cognitive and neurochemical processes underlying decisions under social uncertainty, with implications for research in risk-taking behaviors in healthy and clinical populations.

## Introduction

Coping with uncertainty is essential to navigate a dynamically changing world. Previous work has extensively investigated how individuals evaluate gain and losses under different forms of outcome uncertainty, such as ambiguity and risk^1^. However, evidence about how individuals make decisions under social uncertainty, i.e., when decision outcomes depend on another person, is still inconclusive^2^. Making decisions under social uncertainty is particularly relevant for a vast array of social behaviors. For instance, attempts to detect a partner’s trustworthiness can be interpreted as attempts to reduce uncertainty by predicting the partner’s behavior^3^, suggesting that many social behaviors are a form of risk-taking behavior^4^. Further, findings from the literature on strategic uncertainty that studies decisions under social uncertainty in coordination games seems to suggest that strategic uncertainty can be reduced to individual risky choices like those made when playing lotteries^5–7^.

However, evidence on risky decision-making in social contexts is mixed. For instance, subjective reports on risk preferences for financial stakes and survey-based risk attitudes are not associated with trusting behaviors^8–11^. Moreover, lottery risk preferences do not have any explanatory power of trusting behavior and, if anything, individuals have been observed to be more willing to trust a partner who reciprocates frequently than to trust a gamble with a slot machine that rewards with the same probability^9,12,13^. Further, social contexts make people more likely to engage in risky behaviors and make riskier choices such as during peer interactions^12–16^.

At least two factors need to be considered when studying risky choices under social uncertainty. One the one hand, during social interactions positive and negative consequences of decisions and actions are weighed differently. Riskier decisions in peer interactions could, for instance, be traced back to higher valuation of positive information or expectancy of more positive outcomes, as individuals have been shown to more strongly weight the benefits than the costs of risky behaviors in social contexts^17^. On the other hand, investigations on social behaviors such as trust show that individuals are also less willing to interact with untrustworthy partners who reciprocate infrequently than risk a lottery that rewards with the same probability or randomly determines outcomes^13,18^. This suggests that individuals are more risk-averse in social interactions if there are reasons to believe that the partner may have malevolent intentions, implying a so-called ‘betrayal cost’^18–20^. In particular, when untrustworthy behavior is incentivized, individuals believe a very small proportion of partners will be willing to reciprocate^21,22^ but have a more trustworthy attitude if there are no ostensive reasons for the partner to behave selfishly and exploitatively^23^.

The neurotransmitters involved in these kinds of behavior are not entirely known. Previous work has, for example, investigated the role of the neuropeptide oxytocin in social interactions but did not find significant relationships with trusting behaviors^24,25^. Serotonin is thought to play an important role as well, but the extant literature on serotonergic modulations of expected values under uncertainty is inconsistent. In particular, modulation of the serotonergic system has been proposed to impact the salience of bad outcomes^26^. While depletion of tryptophan, a serotonin precursor, seems to increase aversion to future losses^27^, it also enhances risk-seeking when choosing between losses in a gambling task and reduces it when choosing between gains^28^. Moreover, previous work has suggested that serotonin depletion reduces cooperative behavior despite reciprocal behavior of the partner^29^. Chronic administration of citalopram (a selective serotonin reuptake inhibitor) has been associated with increased valuation of generous offers^30^, suggesting that increased serotonin levels boost the expected values of decision outcomes. However, whether and how serotonin impacts acceptance of potential gains and losses under social uncertainty is yet unknown.

Here, we investigated people’s willingness to accept potential gains and losses under two different sources of uncertainty (namely, social and nonsocial or probabilistic uncertainty), and serotonin manipulation. In two different studies of male samples, we used a within-subject, double-blind, placebo-controlled design, and employed a modified gambling paradigm where decision outcomes were determined by another participant or chance. We controlled for gender during recruitment because of gender-differences in serotonergic effects on social behaviors^31^. We investigated whether individuals are more likely to accept gambles in social or nonsocial contexts and how serotonin modulates acceptance behavior of gambles under these different forms of uncertainty. In particular, participants should be less averse to potential losses in the social condition, thereby increasing acceptance especially for gambles with relatively lower gains and higher losses. On the contrary, serotonin administration might lead to an overall increased acceptance of gambles.

## Materials and methods

### Participants

Thirty-tree (age: 24.3 ± 3.4, Mean ± SD) and 26 male participants (23.3 ± 4.0) were recruited for Study 1 and Study 2, respectively. In Study 1, one participant did not show up for the second session and another was excluded due to the technical problem in the experimental procedure, leaving a total of 31 participants for analyses (24.4 ± 3.5). In Study 2, two participants did not show up for the second session and one stopped the experiment because feeling sick in the second session, leaving a total of 23 participants for analyses (23.6 ± 4.1). Finally, in Study 2, only 4 participants reported mild symptoms following serotonin administration (e.g., feeling sleepy and lack of concentration). Exclusion criteria were as follows: present or past neurological and psychiatric disorders, pharmacological medication up to 2 weeks prior to the study (including participation in other pharmacological experiments), current physical or mental stress and other severe health complications. All participants had normal or corrected-to-normal vision. Finally, only for some of our participants we were able to collect body-mass index (BMI) information. From the datapoints we have, we can infer that our participant’s BMI was within the normal range (23 ± 1.8).

The study was approved by the local Ethics Committee of the University of Lübeck, all methods were performed in accordance with the relevant guidelines and regulations, and participants provided written informed consent for participation. Participants were recruited via flyers on the University campus and the University participant pools. Participants were mostly students or associated with the University. Participants received 8€/h. Moreover, depending on their choices in the gambling task, they could earn an additional monetary bonus. As serotonin has been reported to have differential effects on trusting behavior on women and men, we have focused in this first work on male participants^31^.

### Experimental procedure

Participants took part in two sessions 13–14 days apart. In each session, participants received either citalopram or placebo in a randomized order across subjects according to a double-blind design (see Supplementary Information). After signing the consent form, participants received citalopram (30 mg) or placebo in a white capsule and were instructed to take the pill with a white medication spoon. Afterwards, participants waited 2.5 h in the laboratory to allow for peak serotonin concentrations following previous work^32^. After the waiting period, participants underwent a battery of tasks, among which there was the risk task. Procedures of Study 1 and Study 2 were exactly the same with the difference that Study 1 involved an fMRI session prior to the task. Participants were debriefed at the end of the second session.

To improve credibility of the social condition, participants were told that two roles were possible in the task and that their instructions will depend on the role assigned. Participants were told that role assignment depended on a random ball-drawing procedure that was performed right before the task. Thereby, they drew a ball from a lottery box and were asked to insert the letter on the ball into the computer. Finally, the corresponding instructions were shown, which always described the “gambler” role for all participants in our sample.

**Figure 1:**
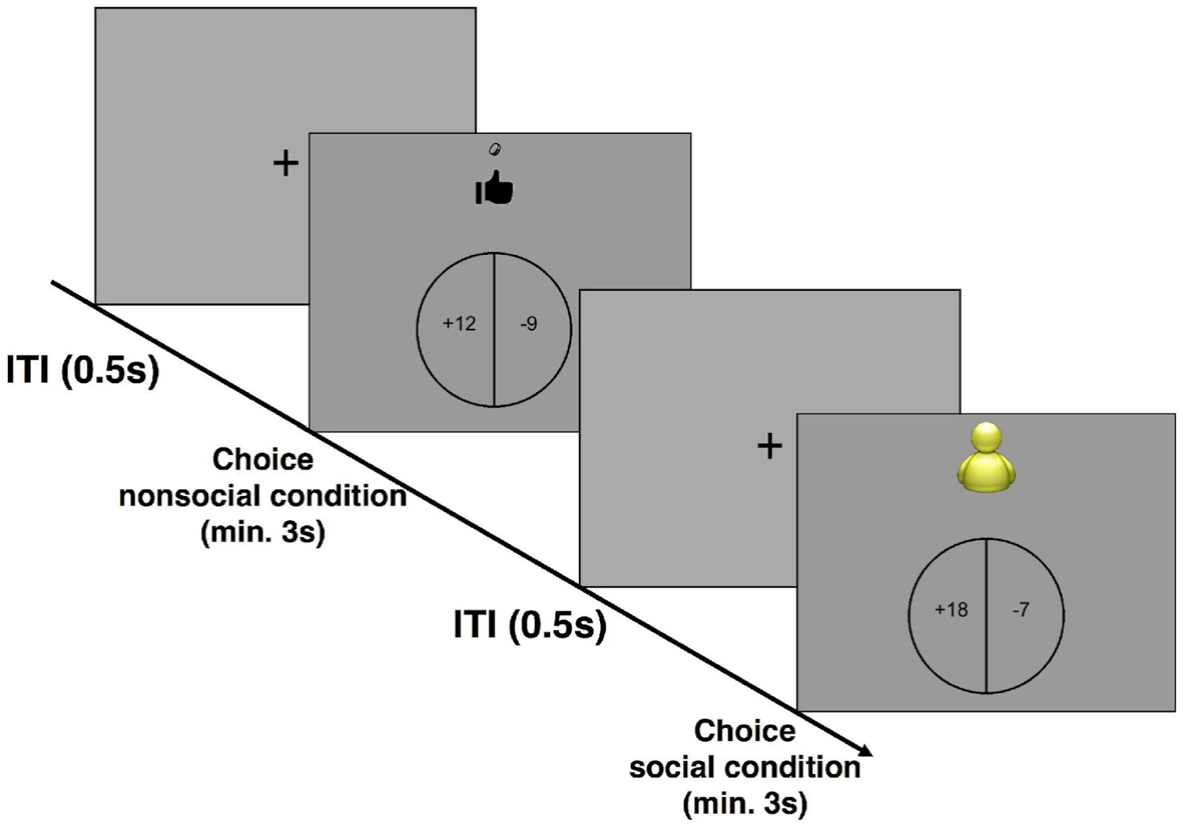
Behavioral paradigm. Timeline of the behavioral paradigm. Participants had to decide whether to accept or reject a gamble with depicted gains and losses. Determination of current gamble’s outcomes was depicted above the gamble. A coin-tossing hand indicated that the gamble’s outcome was decided by the participant with a coin toss (nonsocial condition). A manikin indicated that the gamble’s outcome was decided by another participant (social condition).

During the risk task, participants were presented with different gambles in each trial (Fig. 1). Each gamble implied a combination of a gain and a loss. Above the gamble, two different symbols were presented: either a hand tossing a coin (nonsocial condition) or a manikin (social condition). Participants were told that they had to decide whether they wanted to accept or reject the gamble and that at the end of each session, one accepted gamble for each condition was going to be randomly chosen and actually realized. Gamble’s outcomes with the coin-tossing symbol were decided by a coin toss performed by the participants at the end of the task (nonsocial condition). Gamble’s outcomes with the manikin symbol were decided by another participant at a later time point who received the “chooser” role (social condition). It was made clear that choosers had neither incentives nor deterrents to make any of the two possible decisions, thereby inducing a form of indeterminacy about the choosers' motives that was supposed to yield the highest form of social uncertainty. Both symbols were presented for each gamble in a randomized order. Hence, potential gains and losses were exactly the same in both conditions, allowing us to directly compare participants’ value computations under different forms of uncertainty.

Participants had no time limit for their answers. However, each gamble was presented for at least 3 s to encourage participants to reflect on the gambles and to avoid fast and automatic answers. Between gambles, an intertrial interval of 0.5 s was presented. Possible gains ranged from €10 to €30 (in €2 increments) and possible losses ranged from €5 to €15 (in €1 increments) for a total of 121 trials (1 for each gain–loss combination) for each condition (i.e., social and non-social, resulting in 242 gambles in total per session). For the above-mentioned range of possible gains and losses, we followed a previous study that has shown it to induce reliable loss aversion in participants^33^. Stimuli were presented using Psychtoolbox 3 (http://psychtoolbox.org) on MATLAB 2016b (https://www.mathworks.com).

### Analyses

Hierarchical Bayesian logistic regression models were fit to participants’ acceptance behaviors in the gamble task. The full Bayesian models consisted of three fixed-effect regressors capturing the experimental conditions of the study design, their two- and three-way interactions, and a regressor parameter controlling for subjects’ age with a subject-specific random intercept to account for interindividual variability in acceptance behavior. For Study 1, the priors assigned to the regression parameters were gaussian and uninformative $\beta \sim {\mathcal{N}}\left( {0,10} \right)$. For estimation of regression parameters in Study 2, pointwise posterior model estimates from Study 1 (mean and standard deviation) were used as priors. Letting ${\varvec{y}}_{j}$ be the $T \times 1$ response vector of subject *j* with an observation (response) for each time *t* (where $T$ equals to the total number of observations per subject, namely, 484 trials), the full model in both Study 1 and Study 2 can be compactly written in matrix notation as follows:

$$y_{j} \sim Bernoulli\left( {logit^{{ - 1}} \left( {b_{{0j}} + \beta _{0} + {\mathbf{\upbeta X}}_{j} } \right)} \right)$$

where $b_{0j}$ is the subject-specific varying intercept for subject *j*, $\beta_{0}$ is the fixed intercept, ${{\varvec{\upbeta}}}$ is a $q \times 1$ vector of fixed slopes where $q = 8$ for the eight model regressors, and ${\mathbf{X}}_{j}$ is the $T \times q$ design matrix with regressor values for subject *j* at each time *t*. In particular, regressors coded for the treatment (1 = citalopram; 0 = placebo), social condition (1 = social condition; 0 = nonsocial condition), the value difference of the gamble presented on a given trial, the two- and three-way interactions among these three regressors, and a regressor for subject’s age. Models were fit using Stan with the no-U-turn sampler for efficient exploration of posterior estimates (https://mc-stan.org). Four chains were run with 1000 tuning steps and 20,000 samples each. Visual inspection of the traces and the Gelman-Rubin statistics ($\hat{R}$) were used to assess convergence^34^. In particular, it was checked that model parameters had a $\hat{R}$ value lower than 1.1. For both studies, all model parameters met this criterium, indicating good convergence. Averages of parameters’ posterior distributions were used as point posterior parameter estimates. Parameters with 89% highest posterior density (HDI) intervals that did not contain zero were deemed to be statistically significant^35^. Standardized model parameters were reported for comparisons of the magnitude of the effects. To maintain coherence with binary input variables, we standardized variables by dividing by 2 standard deviations rather than 1^36^. Bayes factors were computed with bridge sampling by estimating evidence in favor of a reduced model ($M_{1}$) without a regressor of interest over the full model ($M_{0}$)^37,38^:

$$BF_{10} = \frac{P(Reduced \,Model|D)}{{P(Full \,Model|D)}} = \frac{{P\left( {D{|}M_{1} } \right)P\left( {M_{1} } \right)}}{{P\left( {D{|}M_{0} } \right)P\left( {M_{0} } \right)}}$$

## Results

### Increased acceptance for lower values under social uncertainty

**Figure 2:**
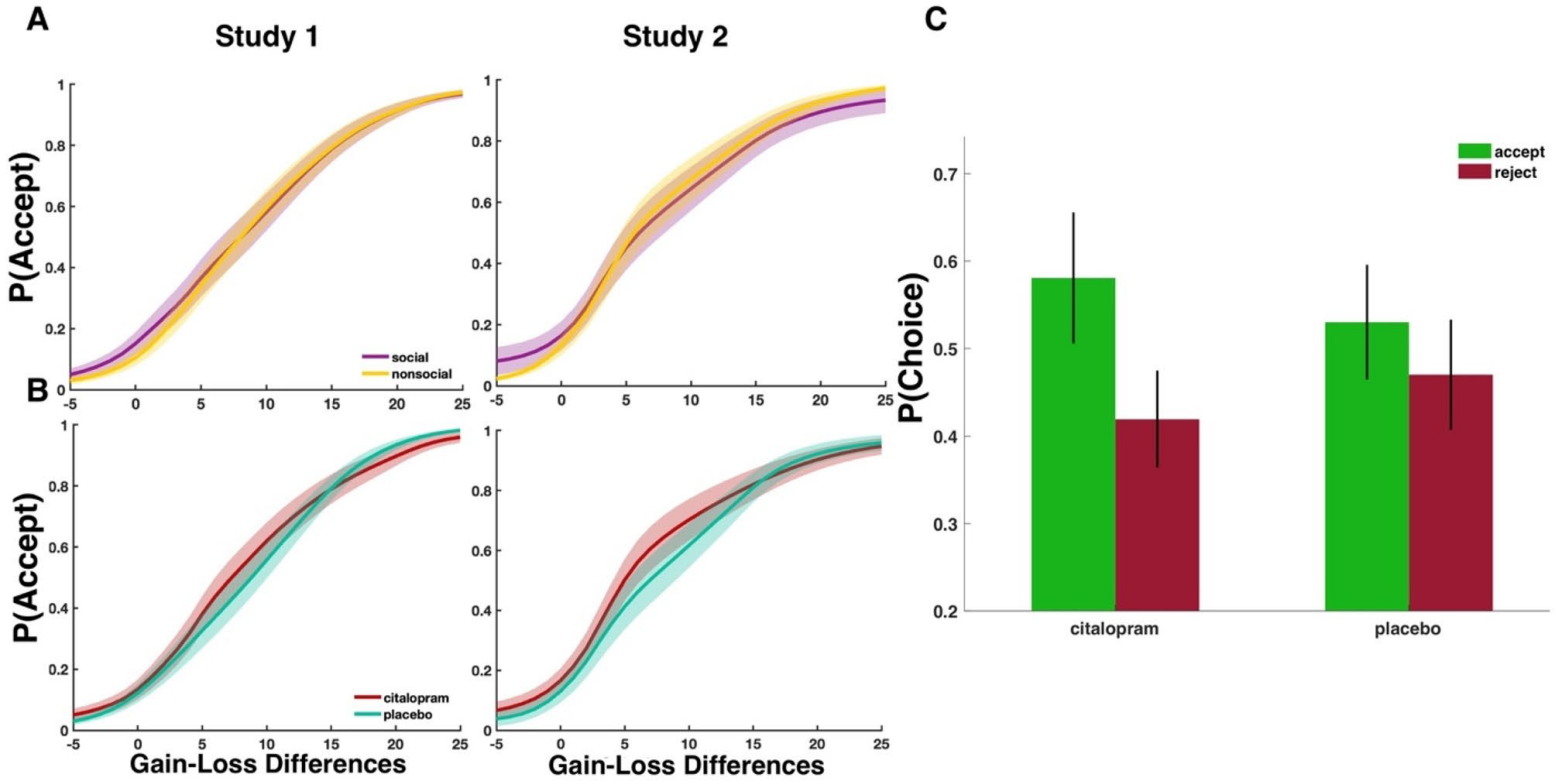
Effects of social uncertainty and serotonin on acceptance behavior. (**A**) Under social uncertainty, participants were more likely to accept a gamble of smaller and negative value differences in Study 1 and Study 2. P(Accept) is the probability of accepting a gamble. (**B**) Citalopram administration increased the likelihood of gamble acceptance especially for smaller value differences in Study 1 and Study 2. (**C**) Average acceptance and rejection behavior for citalopram and placebo.

Results show that individuals were overall more willing to accept gambles in the social condition as opposed to the nonsocial condition (pointwise posterior estimate $\beta$ = 0.35 with standardized estimate (0.04), standard deviation of the posterior probability, SD = 0.13; Table 1). In particular, we found higher probability of accepting gambles of lower outcome value differences in the social condition than in the nonsocial condition ($\beta$ = − 0.03 (− 0.35), SD = 0.01; Fig. 2A and Table 1), leading to a heavy-tailed distribution on the negative end of the considered range of outcome values (Fig. 2A). However, there was not strong evidence for a full model with both the social condition (BF_10_ = 0.976) and the interaction term with outcome value differences (BF_10_ = 1). Nonetheless, in Study 2, we replicated both these effects (main effect of social condition: $\beta$ = 0.22 (0.03), SD = 0.07; interaction effect of social condition: $\beta$ = − 0.04 (− 0.03), SD = 0.01; Fig. 2A) and found not only more convincing evidence for a model with a social condition term (BF_10_ = 0.362) but also strong evidence for a model with an interaction term between the social condition and outcome value differences (BF_10_ = 0.020). These results suggest that when the source of uncertainty was another person, individuals were more willing to accept gambles with smaller gains and larger losses.

**Table 1:** Mixed-effects Bayesian regression model.

Regressors	Study1 N = 31	Study 2 N = 23
Intercept	− 1.88 (2.25)	− 2.00 (0.32)*
Treatment (Tr)	0.62 (0.13)*	0.46 (0.07)*
Social condition (S)	0.35 (0.13)*	0.22 (0.07)*
Value difference (VD)	0.30 (0.01)*	0.30 (0.01)*
Tr * S	− 0.12 (0.17)	− 0.03 (0.1)*
Tr * VD	− 0.02 (0.01)*	− 0.03 (0.01)*
S * VD	− 0.03 (0.01)*	− 0.04 (0.01)*
VD * S *Tr	0.003 (0.02)	0.01 (0.01)
Age	− 0.04 (0.09)	− 0.01 (0.02)

Point posterior parameter estimates (mean) and posterior parameter estimate uncertainty (standard deviation) for the Bayesian mixed-effects logistic regressions of the two studies. Treatment (Tr) codes for the citalopram (1) and placebo (0) session. Social condition (S) codes for the social (1) and nonsocial (0) contexts. VD = difference of the gamble’s values.

*Posterior parameter estimates for which the 89% highest density interval does not include zero.

### Serotonergic enhancement of outcome values

**Figure 3:**
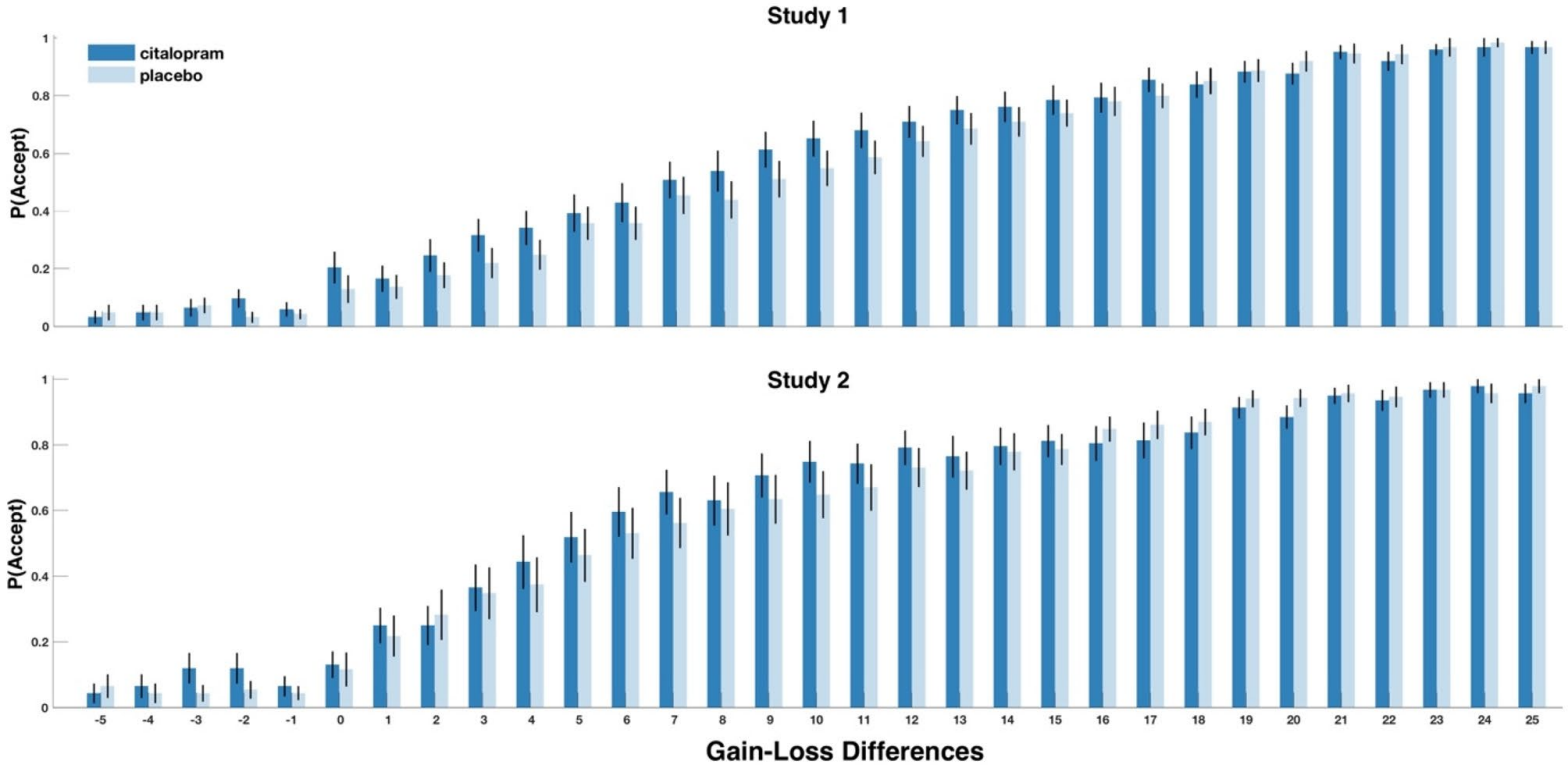
Greater acceptance of gambles of smaller outcome values under serotonin. Gambles with lower outcome value differences were on average more likely to be accepted after citalopram administration as compared to placebo. P(Accept) is the probability of accepting a gamble in Study 1 (above) and Study 2 (below).

In both studies, we observed that participants were more likely to accept a gamble under serotonin than placebo (Study 1: $\beta$ = 0.62 (0.39), SD = 0.13; Study 2: $\beta$ = 0.46 (0.19), SD = 0.07; Fig. 2B,C and Table 1) with strong evidence for this effect in Study 2 (BF_10_ = 0.042). Further, we did not find evidence for a dependency of this effect on the source of uncertainty as indicated by the nonsignificant interaction effect between citalopram administration and social condition in both studies (Table 1) and the stronger evidence for a model without the interaction term between citalopram administration and social condition especially for Study 1 (Study 1: BF_10_ = 445.986; Study 2: 2.140). These results suggest that serotonergic modulation of acceptance behavior is similar under both social and probabilistic uncertainty. Further, like the previous social condition effects, serotonin particularly increased an individual’s willingness to accept gambles of lower outcome value differences especially for Study 2 (Study 1: $\beta$ = − 0.02 (− 0.23), SE = 0.01, BF_10_ = 1.024; Study 2: $\beta$ = − 0.03 (− 0.02), SE = 0.01, BF_10_ = 0.032; Fig. 3 and Table 1), providing evidence for a serotonin-induced increase in risky choices. Hence, serotonin administration affected acceptance behavior in a fashion that depended on the magnitude of the gains and losses of the gamble outcome. However, the increase in acceptance behavior was most evident for small but positive outcome values (i.e., the middle range of the considered value differences; see Figs. 2B and 3). Finally, the serotonergic effect was also context-independent, influencing gamble decisions under both social and probabilistic uncertainty.

## Discussion

We investigated the effects of social outcome uncertainty and serotonin on an individual’s acceptance of potential losses and gains. Our results show that participants were more likely to accept gambles of lower outcome values under social uncertainty, suggesting a positive shift in the weight of gains and losses on choices whose outcomes hinge on another person’s decision. A similar increase in the likelihood of gamble acceptance was found after citalopram administration. However, higher serotonin levels were also associated with overall greater acceptance of gambles irrespective of the current gamble’s source of outcome uncertainty. These results indicate contextual and neurochemical modulations of acceptance behaviors under social and probabilistic uncertainty.

First, we observed that participants were more willing to accept a gamble whose expected value was closer to zero, and even negative, under social uncertainty. These findings contrast previous evidence that individuals are less likely to take risks in social contexts. For example, individuals have been shown to be more risk-averse when payoff outcomes are dependent on another's trustworthiness^23^ and such reduced propensity to take risks has been linked to a betrayal cost (or betrayal aversion) evoked in interactive situations^20^. Experimental settings in which such behavior has been observed, however, were characterized by inductions of negative expectations about the social interaction due to incentives for the partner to defect and behave strategically^39–41^.

In our study, we demonstrated that when such concerns are lifted and the partner has no motivation to behave selfishly, individuals are more willing to make themselves vulnerable in choices whose outcomes depended on the partner. These results are consistent with the idea that individuals have a strong prior about others’ compliance with social norms that foster prosocial behaviors and cooperation across different situations if no other reasons jeopardize such a compliance^42,43^, and concur with previous empirical evidence showing that when temptations for a partner to defect are minimized, individuals are more likely to rely on them^44^. Moreover, they point to the importance of taking into consideration an individual’s expectations when investigating decisions under uncertainty in social contexts. In particular, behaviors in social contexts rely on additional heuristics beyond the economic weighting of costs and benefits, triggering behavioral patterns that largely root in social evaluations of others’ choices, behaviors and personality^45^. This explains why misperception of peer norms contributes to risk-taking behaviors^46^ and riskier behaviors are fostered by knowledge of risky decisions of others^47^. Hence, people are rightly more cautious in their decisions and less willing to make themselves vulnerable, when they have reasons to believe that an actual harm may follow from someone else’s actions. When, however, the conditions to infer malevolent intentions are not given (or attenuated), people are not neutral with respect to the most likely behavior of their peers, as they assume that others are permissive and benevolent, corroborating a willingness to accept vulnerability^41^.

Similar considerations can be made with respect to strategic uncertainty, which often occurs in competitive, social settings. Interestingly, a previous study^48^ that compares strategic uncertainty in a competitive, social setting with probabilistic uncertainty in lotteries, and used a similar modeling approach as in this paper did not observe any across-condition differences in the indifference points of the regression lines (a measure of direct exploration, see for instance,^49^). However, similar to our studies, they observed reduced asymptotes at values close to the extremities (e.g., 0 and 1), giving rise to heavy-tailed distributions that have previously been associated with uncertainty-guided exploration^50^. Importantly, their effect was in the exact opposite direction as ours, with participants being less likely to accept riskier gambles under social as compared to probabilistic uncertainty, likely due to the competitive nature of their paradigm^48^. This suggests that people’s expectations about others might have far-reaching effects on other important decision-making processes such as the usage of different exploratory strategies.

Hence, the cognitive mechanisms underlying evaluations of future outcomes might be rather different in social and nonsocial contexts. In particular, the positive, as opposed to the negative, consequences of a decision might be weighted more strongly in social interactions due to more positive expectations about others, concurring with previous work reporting more positive valuation of risky behaviors in groups^17^. The greater frequency of risky behaviors in peer and group interactions might thus be traced back to a positivity bias reducing the weight negative consequences have in people’s decisions, and increasing the contribution of positive, social benefits^51,52^. This bias likely takes the form of a higher probability (expectancy) associated with positive than negative outcomes during expected value estimations. A promising avenue of future work is whether such bias in people’s social expectations is justified by the evidence they gather from interactions with others, to which degree it can be overwritten, and what consequences this has.

Importantly, in our studies participants knew that their partner did not have any specific incentives or deterrents that would favor undertaking a specific behavioral strategy. We believe that this might have induced a form of indeterminacy about the partner's motives that yielded the highest level of social uncertainty. One possible objection might be that the absence of incentives for selfish and uncooperative behavior implied a shift in one's expectations that left no room for uncertainty. Indeed, our experimental paradigm has likely shifted participants’ expectations toward a more positive outlook on decision outcomes in the social condition, as our participants were not just only more likely to accept gambles under social than probabilistic uncertainty, but also particularly so for gambles with outcome value differences closer to zero or negative. This is quite remarkable, as in nonsocial contexts, similar decisions, such as decisions under ambiguity, tend to be less likely than under probabilistic uncertainty^1^. However, this still does not imply that our experimental design made our participants less uncertain about the decision outcomes of gambles in the social condition. For the absence of incentives for a partner's behavioral strategy does not equal to absence of uncertainty about the partner's behavior. On the contrary, indeterminacy about another person's motives is supposed to yield the highest level of uncertainty about the other's behavior. Despite this feature of our experimental design, it cannot be excluded that people might indeed have employed different mechanisms to resolve such uncertainty, for instance, by retrieving memories about previous social interactions. Future studies need to test how motivational indeterminacy is interpreted by decision-makers and to which degree different types of additional information about another person's motives decrease one's uncertainty about that person’s behavior.

Second, we observed an increase in acceptance behavior induced by citalopram administration. These results are consistent with evidence that tryptophan depletion enhances the salience of bad outcomes reducing risky choices^27^, and that tryptophan supplement reduces loss aversion altering the weighting of gains and losses^53^. In particular, we observed that serotonin administration increases acceptance of gambles of smaller values with a pronounced increase of acceptance for gambles whose outcome value difference lay in the intermediate range of the considered value scale. This suggests that serotonin might modulate risk-taking behaviors as a function of the magnitude of losses and gains, thereby explaining previous mixed results^54^.

Finally, we did not find any interaction effects between the source of uncertainty (social/nonsocial) and serotonergic modulation of choice behavior in the two independent studies. In particular, Bayesian analyses suggest overwhelming evidence for a model without an interaction between citalopram administration and the source of uncertainty. These findings do not seem to align with previous work indicating serotonergic effects of valuation of social behaviors and relationships. For instance, citalopram intake increases evaluation of mutual trust in relationships^55^ and heightens supportive speech toward withdrawn individuals^56^. Such absence of modulatory effects of serotonin in our task may relate to the fact that our participants were unaware of any incentives for the partner to behave selfishly or malevolently. Because this might have led individuals to have positive expectations that elicit behaviors similar to those evoked by overt favorable intentions of the partner^23,57^, a further enhancement of such behaviors might have been difficult to achieve with an acute administration of serotonin. Future studies might, hence, test the opposite prediction, that is, whether serotonin depletion reduces acceptance behavior of gambles under social uncertainty in contexts in which individuals have positive expectations of their social partners. Allegedly, this should produce behaviors similar to the ones observed when participants have reasons to believe in a partner’s malevolent intent.

Another reason for the nonsignificant interaction effect between serotonergic administration and the source of uncertainty might lie in the anonymous nature of the social interaction in our paradigm. For instance, previous work has provided evidence that there are important neural differences between trust in strangers in anonymous interactions and trust in known others in less anonymous contexts^58^. Hence, future studies are needed to investigate whether our results hold in other social contexts with varying degrees of anonymity and forms of social relationships.

A couple of limitations have to be addressed. First, given gender differences in the serotonergic effects on trusting behaviors^31^, our sample consisted of exclusively male participants. Having only male participants helped us exclude gender-related differences in choice behavior, as an investigation of such differences was not the focus of this study. However, it importantly limited the generalizability of our results. Similarly, the absence of BMI information from all of our participants precluded us the possibility to control for variation in BMI, which might have induced additional variability in how effective our fixed citalopram dose was. Nonetheless, thanks to the second study, we were able to replicate our main findings, which strengthens the evidence of the observed effects in male subjects. Future studies are still needed, though, to test whether similar results hold in more heterogeneous samples with bigger sample sizes.

Taken together, our studies reveal that acceptance behaviors under uncertainty are modulated by both the source of outcome uncertainty and changes in the serotonergic brain system. These results provide novel insights into the cognitive and neurochemical processes underlying decisions under different forms of uncertainty that will inform future research in risk-taking behaviors across age and social contexts, such as risk-seeking in adolescents or risk avoidance in anxiety.

## Supplementary Information

Supplementary Information.
