# Supplementary material for: Influences of social uncertainty and serotonin on gambling decisions
Source: Sci Rep. 2022 Jun 17;12:10220. doi: 10.1038/s41598-022-13778-x (PMC9205937; doi:10.1038/s41598-022-13778-x)
Supplement: Supplementary file 1 — Supplementary Information. [file 41598_2022_13778_MOESM1_ESM.docx]

**Experimental Protocol and Instructions**

In both studies, participants arrived at the institute where they were received by the experiment, conducted to the experiment room and instructed about the study procedures. Afterwards, participants signed the consent form for the experiment. In Study 2, they further signed the consent form for the MRI investigation. The whole procedure lasted around 20 minutes.

Afterwards, the treatment procedure was employed. Participants received citalopram (30 mg) or placebo in a white capsule and a white medication spoon whit which they should have brought the pill to their mouth without touching it, to avoid participants might have guessed the content of the white capsule via the sensory feedback received by touching. Importantly, in both studies, a double-blind procedure was implemented, hence the experimenter was also blind to the content of the white capsule. During the waiting period of 2.5 hours in the laboratory (necessary for peak serotonin concentrations), participants were allowed to engage themselves as they pleased. The only restriction was that they had to wait in the experiment room where the experimenter could monitor them in case of any symptoms following citalopram administration. Toward the end of the waiting period, participants completed questionnaires about their current state and feelings.

After the waiting period, participants underwent a battery of tasks. In Study 1, participants underwent the risk task, a jumping-to-conclusion task, the trust game and a facial evaluation task. Study 2 was very similar, with the difference that before the risk task, participants completed a “social” risk task in the MRI scanner.

Before the risk task, a ball-drawing procedure was implemented. Participants were told that there were two roles in the task, the “gambler” role and the “chooser” role. Further, they were told that task instructions depended on the role they were going to be assigned to. The ball-drawing procedure was meant to determine their role. Thereby, they drew a ball from a lottery box and were asked to insert the letter on the ball into the computer. Participants saw on the screen instructions reporting that based on the letter drawn (and shown on the screen), they were going to play as “gambler” in the risk task and were going to be matched with a future participant taking part in the experiment at a later time who was going to play the same task as “chooser”.

Next, participants were instructed that during the risk task, they were going to be presented with different gambles in each trial. Each gamble implied a combination of a gain and a loss. Above the gamble, two different symbols were going to be presented: either a hand tossing a coin (nonsocial condition) or a manikin (social condition). Participants were told that they had to decide whether they wanted to accept or reject the gamble and that at the end of each session, one accepted gamble for each condition was going to be randomly chosen and actually realized. Gamble’s outcomes with the coin-tossing symbol were going to be decided by a coin toss performed by the participants at the end of the task (nonsocial condition). Gamble’s outcomes with the manikin symbol were going to be decided by the “chooser” (social condition). Choosers did not have either incentives or deterrents for their decisions.

Participants were told that the gambles were presented for at least 3 seconds and were asked to provide an answer within this time frame. If they answered within this time frame, they were allowed to proceed after the end of the 3 seconds. Otherwise, they were told they proceeded after they made a decision. Participants were told that gains were going to be paid out, while losses were going to be deducted from their overall payoff. To improve credibility of the social session, the following instructions were added. Given that the experimenter had to wait for the matched “chooser” to make a decision about participants’ gains and losses, participants were informed at the beginning of the first session (and reminded at the beginning of the second session) that they were not going to receive their payoffs paid out after completion of the study, namely, at the end of the second session. They were told they had to wait a couple of days instead before their monetary payoffs could be transferred to them.

At the end of the risk task in both sessions, participants saw the gambles that were randomly selected to be played out for the social and nonsocial conditions. If the gamble for the nonsocial condition was accepted by the participant, the experimenter gave them a coin and the participant actually played the gamble by coin flip. If the gamble for the social condition was accepted by the participant, the experimenter recorded the gamble and told the participant that the outcome of the gamble will be subsequently decided by a participant in the role of “chooser” who was going to participate in the same experiment at a later timepoint.

At the end of each session, participants had to undergo a post-experiment monitoring phase for detection of possible side-effects following citalopram administration. Participants were allowed to leave the laboratory around 7 hours after drug administration and were strongly advised against driving back home by themselves. They were instead asked to use public transportation or be picked up by friends/family. At the end of the second session, participants were debriefed.
